# Establishing radiation therapy treatment planning effects involving implantable pacemakers and implantable cardioverter‐defibrillators

**DOI:** 10.1120/jacmp.v11i1.3115

**Published:** 2009-12-23

**Authors:** Michael S. Gossman, Alison R. Graves‐Calhoun, Jeffrey D. Wilkinson

**Affiliations:** ^1^ Tri‐State Regional Cancer Center Medical Physics Section Ashland Kentucky USA; ^2^ Medtronic, Inc. External Research Program Mounds View Minnesota USA

**Keywords:** defibrillator, ICD, implantable, pacemaker, radiation, TG‐34

## Abstract

Recent improvements to the functionality and stability of implantable pacemakers and cardioverter‐defibrillators involve changes that include efficient battery power consumption and radiation hardened electrical circuits. Manufacturers have also pursued MRI‐compatibility for these devices. While such newer models of pacemakers and cardioverter‐defibrillators are similar in construction to previously marketed devices – even for the recent MRI‐compatible designs currently in clinical trials – there is increased interest now with regard to radiation therapy dose effects when a device is near or directly in the field of radiation. Specifically, the limitation on dose to the device from therapeutic radiation beams is being investigated for a possible elevation in limiting dose above 200 cGy. We present here the first‐ever study that evaluates dosimetric effects from implantable pacemakers and implantable cardioverter‐defibrillators in high energy X‐ray beams from a medical accelerator. Treatment plan simulations were analyzed for four different pacemakers and five different implantable cardioverter‐defibrillators and intercompared with direct measurements from a miniature ionization chamber in water. All defibrillators exhibited the same results and all pacemakers were seen to display the same consequences, within only a a±1.8% deviation for all X‐ray energies studied. Attenuation, backscatter, and lateral scatter were determined to be −13.4%, 2.1% and 1.5% at 6 MV, and −6.1%, 3.1% and 5.1% at 18 MV for the defibrillator group. For the pacemaker group, this research showed results of −15.9%, 2.8% and 2.5% at 6 MV, and −9.4%, 3.4% and 5.7% at 18 MV, respectively. Limited results were discovered from scattering processes through computer modeling. Strong verification from measurements was concluded with respect to simulating attenuation characteristics. For IP and ICD leads, measured dose changes were less than 4%, existing as attenuation processes only, and invariant with regard to X‐ray energy.

PACS number: 87.53.Bn, 87.53.Dq, 87.53.Tf, 87.66.Jj

## I. INTRODUCTION

An external pacemaker was first designed and built by the Canadian electrical engineer John A. Hopps in 1949, utilizing a series of vacuum tubes and an AC wall power supply.^(1)^ This artificial pacemaker, which should not be confused with the heart's natural pacemaker, was used to regulate heart rate declines (bradycardia) through electrical impulses delivered when a generator conducts electricity through electrodes contacting heart muscles. The detection of blood movement and breathing are interpreted, recorded, and processed, where an electrical impulse may then be sent to the electrodes in order to help maintain a normal heart rhythm. Newer devices have multiple circuits (chambers) for connecting leads. This design permits timed electrical stimulation for one or even multiple areas of the heart (i.e. the right ventricle only, both the right atrium and right ventricle, or an atrium and both ventricles).

Twelve years of further research went into the development of a device capable of correcting both for the onset of arrhythmia and ventricular fibrillation. The new device, invented by Nobel Peace Prize recipient Bernard Lown in 1961, was capable of delivering an impulse in less than 20 seconds.^(^ ^2^ ^,^ ^3^ ^)^ Initially referred to as the cardioverter, it has since become known as the defibrillator.^(4)^ Operating from DC voltage alone, human implantation became possible for it and the pacemaker. Modern day versions of the defibrillator are now able to detect and correct for ventricular tachycardia.

Within the following eight years, advances in these devices became more political and publicized, yet more complex to achieve. Researchers struggled to create a device capable of sending multiple electrical impulses and providing different levels of electrical shock when required.^(5)^ The aim was to determine if it were possible to design a device that could modulate the impulse sent to increase low heart rates to an acceptable rate (pacing), deliver a mild shock synchronized to an inappropriately fast heart rate (cardioversion), or deliver a much more powerful shock to a heart in ventricular fibrillation (defibrillation).^(6)^ The use of computer algorithms and advancing hardware, pioneered by a team led by Michel Mirowski and Morton Mower, resulted in the first ever implantable cardioverter‐defibrillator (ICD) in 1969.^(7)^

**Figure 1:**
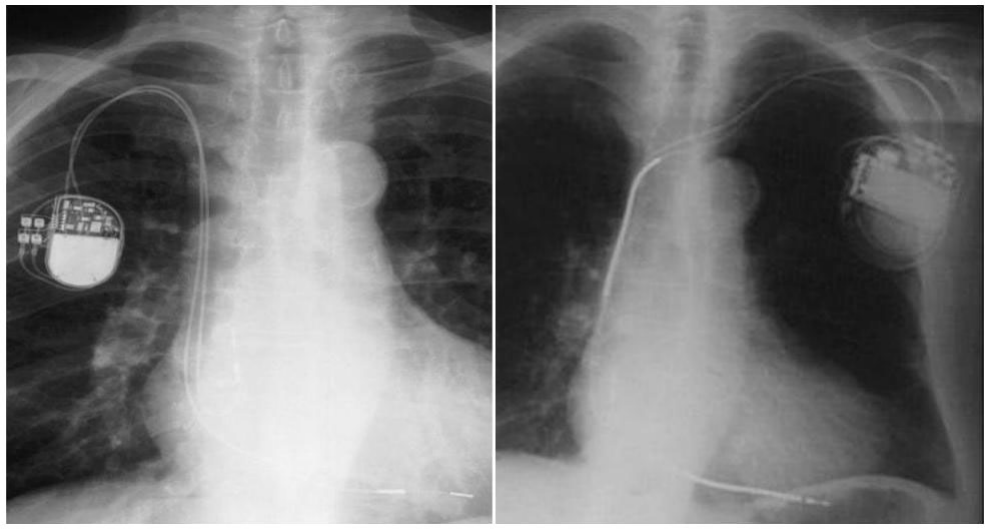
[Left]: An X ray of patient implanted with dual chamber pacemaker Thera‐l model 8968i (most similar to Adapta model ADDR01); [Right]: an X ray of patient implanted with ICD Maximo DR model 7278. Both include right atrial (RA) and right ventricular (RV) electrodes.

Now, forty years later, the implantable pacemaker (IP) and ICD are extremely valuable and commonly used by cardiovascular physicians. There are still, however, some aspects of the use of pacemakers and ICD's that are not well understood. Within the field of radiation oncology, the aim is to utilize scientific techniques available to treat cancer with radiation. When the material being irradiated is not similar to that of a patient – like a metallic pacemaker, for example – the physics of the radiation beam is altered. This presents a challenge on multiple levels. Problems are known to have occurred during in vitro testing.^(^ ^8^ ^,^ ^9^ ^)^ From these observations, clinicians have been made aware of possible interactions with implanted and external devices.^(10)^ Not only is this an issue during computerized tomography (CT) image acquisition initially, but it is an issue for delivering radiation for the treatment of cancer.^(^ ^11^ ^,^ ^12^ ^)^ Figure 1 illustrates the size and location of such devices from a chest X‐ray radiograph.

Simultaneously, engineering tests are now in progress, which are designed to address concerns about the electronic integrity of newer devices in high levels of radiation.^(13)^ It is ideal to obtain a device that is stable, even under a high flux of radiation, while capable of incurring doses far exceeding the current device recommendation of 200 cGy.^(14)^ To this effect, such active investigations should provide a new discussion for dose limitations regarding the electrical stability of these newer devices. Meanwhile, the effects on a radiation beam have yet to be determined.

A patient can present with medical difficulty so as to require the use of an implantable cardioverter‐defibrillator or pacemaker in order to prevent sudden death, and require radiation therapy concurrently to treat local disease. Dosimetric conclusions from device interference in radiation therapy have never been addressed from a treatment planning standpoint. Very recently, the American Association of Physicists in Medicine (AAPM) has issued strong recommendations to the U.S. Food and Drug Administration that operators of radiation machines be trained to look for, identify, and pursue more knowledge about these devices.^(15)^ As a direct result of these concerns, there is now interest in revealing the magnitude of dose change exhibited by a high energy X‐ray beam when such a device is within the field of radiation or in its proximity. Since it is currently not feasible to place detectors inside of a patient at precise enough locations with respect to the cardiac device in order to determine beam altering effects, the most suitable approach is to approximate the outcome by simulation in water. Where electronic functionality and stability are being studied elsewhere, this research presents results from dose delivery simulation using a treatment planning computer and verified ionization measurements, with an extensive set of implantable pacemakers and cardioverter‐defibrillators.^(16)^ This research represents results from 67% of all such cardiac rhythm device models marketed by Medtronic, Inc. and constitutes 32% of all cardiac rhythm device models marketed by any company nationally.^(17)^

## II. MATERIALS AND METHODS

### A. Computerized tomography acquisition

**Figure 2:**
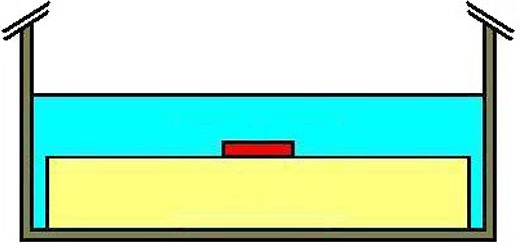
Position of the device (red) during CT acquisition; the acrylic plate and the device are both submerged inside the water tank.

A water phantom having dimension 30cmwide×40cmlong×38cm high defined the scanning volume for simulations. The phantom, a CNMC model WP‐3040 (CNMC Company, Inc. Nashville, TN), is constructed of 3/8” clear acrylic on each of the sides including the white‐papered acrylic bottom. In order to insure adequate backscatter equilibrium, acrylic plates of dimension of 24.8cmwide×24.8cmlong×8.0cm high were placed at the bottom of the tank. The level surface of the acrylic phantom placed within the water phantom further insures that each device will lie flat when positioned on top of the acrylic plates. The WP‐3040 phantom was filled with water until the depth to the submerged acrylic was 5.6 cm. This depth is adequate for both 6 MV and 18 MV X‐ray build‐up. With the ICD or IP on top of the acrylic plates, this distance represents the exact depth to the posterior side of every device studied. For each consecutive scan, a device was identified, labeled, and taped to the hard surface of the acrylic for an identical and easily reproducible geometry. The set‐up for CT acquisition is illustrated in Fig. 2.

**Figure 3:**
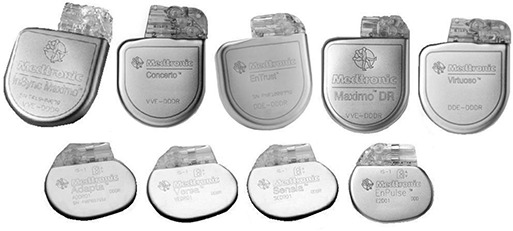
[Top]: Five ICDs [Left to Right]: InSync Maximo model 7304, Concerto model C154DWK (VVE‐DDDR), Entrust model D154ATG, Maximo DR model 7278, and Virtuoso model D154AWG; [Below]: four implantable pacemakers: Adapta model ADDR01, Versa model VEDR01, Sensia model SEDR01, and Enpulse2 model E2DR01.

Computerized tomography was conducted using a GE LightSpeed RT scanner (General Electric, Fairfield, CT) using a protocol for stereotactic radiosurgery scanning. The average technique for the scans was 120 kVp X‐ray energy at 278 mA and 79.7 s scan time in helical mode. A 50 cm diameter circular field of view was used with a couch increment of 1.25 mm/slice. Once the device was imaged successfully, the process was repeated one‐by‐one for all the remaining devices, such that a CT scan was performed for each of the devices separately. The collection of devices used in this study is shown in Fig. 3.

Scan image acquisition was commissioned for use of the extended Hounsfield units (HU) range.^(18)^ Having an extended range for CT unit values allows for a more accurate determination of material density after scanning. It is important to have the correct HU value to represent the material, since the value is directly involved in dose computations by the planning software. The default range for the GE LightSpeed RT scanner is −1,024 to +3,071HU. This differs greatly from its extended range of −31,743 to +31,743HU. A value of well over +3,071HU is expected for metals such as titanium (roughly 8,000 HU) and stainless steel (roughly 12,000 HU). It was seen from scan acquisition that in certain portions of the device, higher end Hounsfield units were evident. Therefore, extended range commissioning was appropriate for involvement, where such results are directly involved in simulated dose computations. Once each scan was reconstructed, all nine independent scan sets containing a total of 2,182 slices were transferred to a treatment planning computer.

### B. Dose simulation: computer modeling

Eclipse external beam planning software version 8.1.20 (Varian Medical Systems, Inc. Palo Alto, CA) was used for the dose computational component of the study. Immediately following scan import, each was visually examined for artifacts on each slice. It has been already seen that high density materials undergoing CT acquisition result in incorrect Hounsfield units in locations surrounding them. This effect was made apparent by visible inspection of the digital reconstructed radiograph (DRR). As with most metals, a beam hardening effect of the initial 120 kVp X‐ray beam results in improper sampling of data made apparent by fan‐shaped streaking through the device. Proper handling of this occurrence eliminates the problem entirely.

Within the contouring workspace of the computational software, each device was studied for the best window and leveling resolution, then contoured on all slices. A new structure was manually created and put through Boolean logic so as to have the exact same volume as the device being studied. A margin for the structure was programmed to increase its size by 3.5 cm. The new structure volume was then redefined using the Boolean tool, such that the resulting volume did not include the volume of the device being studied. The resulting new structure is a volume that envelopes the ICD or IP device entirely, with a margin of 3.5 cm, and excludes the volume of device under investigation. In order to remove any analysis errors caused by metal‐streaming artifacts, this surrounding structure was assigned a CT‐density value corresponding to 0 HU. The surrounding phantom volume thus has a valid water density definition. Every device being studied remained within Hounsfield units that were appropriate for its metallic construction.

The Varian Anisotropic Analytical Algorithm (AAA) version 8.1.20 was commissioned within the planning software for a Varian high‐energy particle accelerator model 21EX with photon energies of 6 MV and 18 MV incorporating Varian Golden Beam data. An output calibration of 1.000 cGy per monitor unit (MU) at the source‐axis distance was performed according to the AAPM Task Group No. 51 protocol.^(19)^ Field definition was assigned at normal positions in accordance with IEC coordinate system 61217 (previously known as IEC 1217) having gantry angle 180°, collimator angle 180° and couch angle 180°.^(^ ^20^ ^,^ ^21^ ^)^ With a point on the phantom surface set as the user origin, the beam isocenter was adjusted to be positioned at the center of the device volume, which was roughly 5 cm deep for all devices.

The reference point for calculation was prescribed to receive 200 cGy at 400 MU/min within the field. In order to insure adequate build‐up for the reference point while also prohibiting scatter contributions which may occur if the point was set too close to each device, the reference point for prescribed dose calculation was positioned off‐axis superiorly from the device at a depth 3.5 cm in the water. Since the largest of the devices, the ICD version InSync Maximo model 7304 with orthogonal face area 7.3cm×5.1cm fits within 3.7 cm (half length) of the central ray along any axis, an off‐axis distance of 8 cm was designated for the final position of the primary reference point in all plans. The radiation field was defined by $30\times 30\;{\text{cm}}^{2}$ jaw collimation, where ideal properties are seen for flatness and symmetry. The smallest possible dose calculation grid of 1.25 mm was assigned.

Eclipse software allows the user to place calculation points of interest on any plane of the three‐dimensional scan. Point placement was determined based on device construction knowledge. Care was taken to place points directly underneath known areas of high atomic number (Z) where the largest dose gradients were expected.

**Figure 4:**
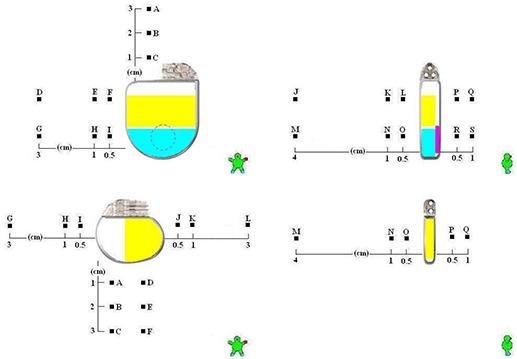
For the Maximo DR model 7278 implantable cardioverter‐defibrillator [Top] and Adapta model ADDR01 implantable pacemaker [Bottom]: (a) [Left]: symmetric lateral calculation points are indicated in the coronal plane; (b) [Right]: calculation points positioned at various depths are shown in the sagittal plane.

For implantable cardioverter‐defibrillators, calculation point placement involved superior distances from the device at intervals 1.0 cm, 2.0 cm, and 3.0 cm. No points were positioned inferior, since the highest density metal is present mostly superiorly. Points positioned laterally were at locations 0.5 cm, 1.0 cm, and 3.0 cm away, both on the superior section of the device and at the inferior section. Points were also set posterior to the device at 0.5 cm, 1.0 cm, and 4.0 cm and anterior to the device at 0.5 cm and 1.0 cm away. These calculation points for the ICD group are set identically as illustrated on top in Fig. 4. The more concentrated areas of high atomic number are colored for convenience of this discussion.

Implantable pacemakers are constructed differently. It is expected that the anterior to posterior dose change will be of less magnitude than it is for the ICD. Instead, more change is expected between the lateral sides. Point placement was not considered superiorly, but considered inferiorly instead, at intervals 1.0 cm, 2.0 cm, and 3.0 cm. Since the higher density metals are present more abundantly on one side, points were allocated bilaterally at locations 0.5 cm, 1.0 cm, and 3.0 cm away. This is opposite to that of the ICD. Points were then set posterior to the device by 0.5 cm, 1.0 cm, and 4.0 cm and anterior to the device by 0.5 cm and 1.0 cm. No distinction was seen as necessary to separate lateral results superior and inferior as done with the ICD. This was determined given the uniform construction of the pacemaker in the superior‐inferior direction. Nonuniformity in construction is obvious laterally only. Calculation points for the pacemaker group are illustrated on the bottom in Fig. 4.

Differences of considerable magnitude are identifiable for some characteristics between computer calculations and measurements. Since computer algorithms for simulating dose consequences are based on advanced mathematical relationships to the media being planned on, measurements are always considered the most accurate scientifically. In comparison to previously published research in a similar study, the AAA computer algorithm was seen to underestimate the magnitude of these characteristics in all areas for both of the X‐ray energies chosen here. In that recognized study, Monte Carlo results detailed AAA algorithm inabilities to emulate precise dosimetric distributions for vascular access ports constructed of titanium.^(22)^ The attenuated dose was within a few percentage points between Monte Carlo and AAA. Backscattering and lateral scattering were even more underestimated. It is noted that a similar pattern of behavior is displayed by the AAA algorithm for this study on pacemakers and defibrillators.

In order to simulate dose involving high atomic number materials (according to AAPM Task Group No. 63), the plan was first calculated using heterogeneity correction.^(23)^ The plan was then copied and recalculated using no heterogeneity correction. The effect of having the device in the radiation beam is identical to the quotient of point‐dose between the density‐corrected plan and the water‐equivalent plan. It was expected that these calculation points would accurately describe the physical consequences of having such a device in a beam of radiation. While anterior points indicate changes in backscatter, posterior points provide information regarding attenuation. Similarly, points positioned laterally away from the device yield results for side‐scatter. Both 6 MV and 18 MV were analyzed for all nine devices using nine independent CT scans with nearly 18 calculation points each. A total of 684 calculation points were analyzed within 36 treatment plans.

Prior to radiation measurement verification, dosimetry from the treatment planning computer was reviewed to determine if there existed any similarities from any of devices for all three characteristics: attenuation, lateral scatter, and backscatter. Nearly identical results for each characteristic were observed for all pacemakers. Defibrillators were found to have nearly equivalent data as well. Therefore, only one device per group was chosen for direct radiation measurement. The irradiation measurement results for only one device per group then provides a reasonable characterization of the entire grouping for comparison with the computer model.

### C. Radiation measurements

Treatment planning system results were verified by direct ionization measurements. The dosimeter system included mini‐thimble PTW chamber model TN31014 (PTW, Freiburg, Germany) with sensitive volume 0.015 cc. The electrometer connected was CNMC model 206 with 200 nC feedback module model 206‐110.

The design set‐up incorporated the use of the large WP‐3040 water phantom and a few CIRS Plastic Water phantom plates (CIRS, Inc., Norfolk, VA), each of size $40\times 40\;{\text{cm}}^{2}$. First, a 5 cm model PW‐4050 plate was placed on the couch of the 21EX particle accelerator for adequate backscatter build‐up. Secondly, a 2 cm model PW‐4020‐518 plate was added. It includes a cavity machined to accommodate the PTW model TN31014 chamber at a depth of 1 cm. Then, the WP‐3040 natural water phantom was positioned on top.

**Figure 5:**
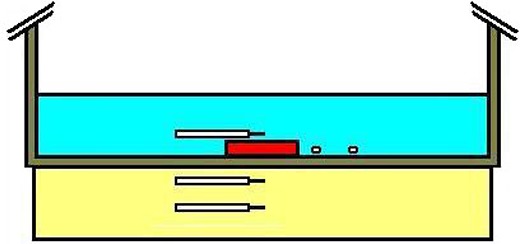
Position of the ionization chamber relative to the device (red) for all five measurements; measurements were facilitated to provide data for attenuation at distances 2.0 cm and 4.0 cm posterior, for lateral scatter on the battery side of each device at 1.0 cm and 3.0 cm away, and backscatter at 0.5 cm anteriorly for both 6 MV and 18 MV X rays.

Ion chamber positioning was conducted to replicate the geometry illustrated in Fig. 4 for each device grouping. The set‐up for measurements is shown in Fig. 5. The ion chamber was inserted into the acrylic plate, as in the illustration, where the resulting depth to the chamber is 2 cm below the inside bottom of the natural water phantom. This is understood by observing that the white‐papered clear acrylic base of the WP‐3040 water phantom is 3/8” thick and the resting chamber underneath is centered in the 2 cm Plastic Water plate. The natural water phantom was then filled to a depth of 5.6 cm, replicating the simulation source to surface distance. The pacemaker or ICD being studied was positioned precisely along the projected central ray of the beam. The quotient of readings with and without the ICD or IP in the beam resulted in the anticipated attenuation and scatter results. This process was repeated for each device grouping at both X‐ray energies stated.

For all measurements, the detector was equilibrated nominally to +300V at the center‐pin. The accelerator was set at the normal position, matching the treatment plan simulation geometry above, and programmed to deliver 200 MU at a rate of 400 MU/min using 6 MV and 18 MV X‐rays. During beam activation, ionization readings were taken posterior from the pacemaker or ICD being studied at the depths 2 cm and 4 cm below the device. It is noteworthy that in order to achieve the 4 cm posterior distance to the chamber, a single 2 cm thick model PW‐4020 plate was added between the WP‐3040 water phantom and the existing chamber plate.

Ionization readings were also taken with the chamber positioned laterally within the plane of the device at distances 1 cm and 3 cm away. To accomplish this, the chamber was rested in the bottom of the large water phantom and taped in position. Finally, readings were then taken at a distance 0.5 cm anterior to the device. When data was taken without the ICD or pacemaker present for backscatter measurements, bolus material was constructed and placed at the bottom of the tank to emulate the position of the device, thus enabling simple placement of the ion chamber at the same source‐detector distance on top of the bolus.

## III. RESULTS

**Figure 6:**
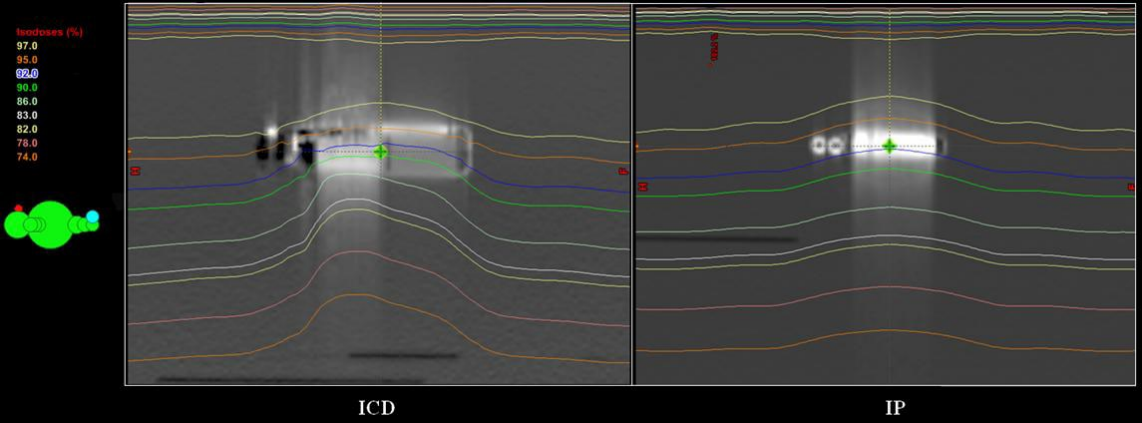
Sagittal view of two plans indicating isodose gradients around a device within an 18 MV X‐ray beam is presented. [Left]: a simulation plan for cardioverter‐defibrillator Maximo DR model 7278; [Right]: a simulation plan for pacemaker Sensia model SEDR01.

Large differences are seen in the treatment planning process between pacemakers and defibrillators with regard to the distribution of dose surrounding them. However, changes in backscattering and lateral scattering were not as significant as the effect of attenuation. The sagittal view of the isodose distribution for a pacemaker and an ICD was captured in Fig. 6 from the treatment planning system. With the electrode connection area shown superiorly for both devices, one may notice the decrease in dose in this area and the dose build‐up inferior to it. This is an expected result, since the connection area is mostly plastic and since higher Z compartments are present more inferiorly. Lateral scattering is less apparent in both views; the local disturbance of the 95% isodose line relative to the device border suggests the presence of lateral scattering.

A posterior dwindling of dose is easily seen beyond the device. It is especially noticeable for the ICD, where the isodose distribution bends asymmetrically around the device. This is indicative of a more diverse design, where construction consists of high density components that vary in density. The least dense area of the ICD is identified as the most inferior region. The attenuation swing is less notable at isodose line 82% for the pacemaker. However, the gradient becomes more conspicuous for the ICD and IP at isodose lines 82% to 78%.

All defibrillators were seen to exhibit the same results and all pacemakers were seen to display the same consequences, within only a a±1.8% deviation for all energies. It was deduced from this information that appropriate representations in the number of devices to measure for comparison against computer simulation involve a single ICD and a single pacemaker. Therefore, only the Concerto model C154DWK (VVE‐DDDR) and Versa model VEDR01 pacemaker were used for measurements. Results from the treatment planning system modeling and radiation measurement from the ionization chamber are presented in Table 1.

**Table 1:** Results from computer simulation and radiation measurement.

*X‐ray Energy*	*Device*	*Characteristic*	*Min*	*Max*	*Min*	*Max*
			*Simulated Results*		*Measured Results*	
6 MV X‐rays	ICD	Attenuation	−5.4%	−9.9%	−8.8%	−13.4%
		Backscatter	−0.1%	−0.2%	2.1%	2.1%
		Lateral Scatter	−0.8%	0.8%	0.0%	1.5%
	IP	Attenuation	−8.2%	−15.2%	−9.2%	−15.9%
		Backscatter	−0.7%	−0.2%	2.8%	2.8%
		Lateral Scatter	−1.5%	0.6%	0.0%	2.5%
18 MV X‐rays	ICD	Attenuation	−3.6%	−6.3%	−2.9%	−6.1%
		Backscatter	−0.4%	0.2%	3.1%	3.1%
		Lateral Scatter	−0.6%	0.7%	0.4%	5.1%
	IP	Attenuation	−5.7%	−9.0%	−5.7%	−9.4%
		Backscatter	−0.6%	0.1%	3.4%	3.4%
		Lateral Scatter	−0.8%	0.4%	0.0%	5.7%

It can be quickly acknowledged that attenuation results are the most significant. The condensed table of data offered is highlighted by attenuation at a level of 15.9% for the IP and 13.4% for the ICD at 6 MV X‐ray energy. Although results for attenuation at 18 MV are noticeably less, the importance of their magnitude is still well understood. Again, a greater amount of beam attenuation is exhibited by the pacemaker than the ICD at 9.4% and 6.1%, respectively. The variance between measured results and simulated results through all energies is noted. Simulated results for the ICD at 18 MV indicate greater attenuation than that measured. Data associated with this physical characteristic of attenuation are similar in general, discovered within ±0.7% between the two methods of analysis. The only outlier for attenuation is the 3.5% difference for the ICD at 6 MV.

While computer simulation results reveal little of significance regarding backscattering and lateral scattering, measurements demonstrate a little more presence from these phenomena. This is identified from numerical comparisons in Table 1. Specifically, backscatter at 6 MV for either device was measured to levels of 3%. The 18 MV beam backscattering was measured to levels of only 2.0%. Together, most of the results fall within a mark of 2.7%±0.7% maximally, encircling all devices and all energies. Lateral scatter at 18 MV was seen as being a little more dominant at around 5.4%, on average, under an 18 MV beam. The average lateral scatter in a 6 MV beam was only 3.0% in comparison.

In contrast to the measured results, the computer‐modeled dose reduction is underestimated in the attenuation process by as much as 3.5% at 6 MV for the ICD group. For scatter processes backward and laterally, an obvious demarcation of dose change from such devices is not present for the AAA. At best, only visual observations can be performed to help the treatment planner qualitatively approximate the likely scenario of dose alteration around the device. In this study, attenuation processes at 6 MV and 18 MV appear to be modeled well by AAA. Processes of scatter were significantly underestimated in comparison to the true magnitude of effects evaluated from direct radiation measurements.

The single wire lead model 5076‐85CM (used for IP pacing) and the triple wire defibrillation lead model 6947‐58CM (used for ICD pacing, right ventricular coil placement and SVC superior coil placement) were both studied through measurements only. Minimal attenuation was determined to exist at a maximum of around 3.8% from both energies at a distance of 5 mm away. Scatter dose increases were not observed. Connections from such leads to the pacing device and to the anatomy of a patient are illustrated in Fig. 1.

## IV. DISCUSSION

Computer treatment planning systems are relied on without debate for the simulation of dose to a patient prior to delivering radiation. Isolated limitations in the use of the newer AAA algorithm are made more readily apparent here.^(24)^ The algorithm has the capability of demonstrating the extremely accurate isodose distributions for tissue‐like bodies on a daily basis for patients.^(25)^ However, such representations are questioned when calculations are applied to items with high atomic number.^(26)^ These algorithms are certainly more reliable when extended Hounsfield unit commissioning has been engaged. Still, even with its use, underestimations remain distinguishable. This research study demonstrated that the isodose distribution properly shifts for attenuation under a pacemaker or defibrillator with high Z components. It also showed how the algorithm accounts for a variance in the shift when the metal components are not uniformly constructed throughout the device. This is not only important for more accurate results from investigations on the effect of high Z materials in the beam of radiation, but also for patients who have high Z prosthetic implants.^(^ ^27^ ^,^ ^28^ ^,^ ^29^ ^)^ These Hounsfield unit values are used directly in the computation of dose by treatment planning systems. Therefore, the software used for calculating dose must also be able to adequately interpret and handle Hounsfield units assigned.

To illustrate this, we consider the devices previously mentioned in Fig. 4. Shown in yellow, the Maximo DR model 7278 ICD battery has a composition that is mostly 29.5% iron (Z=26), 12.6% silver (Z=47) and 11.8% vanadium (Z=23). The battery was determined to exhibit 3,800 HU. Seen in light blue is a high voltage capacitor, visualized in the same plane as the battery. It was determined to have 1,500 HU. Anterior to these two areas, in pink with a circular cross‐sectional shape, is a patient alert beeper. The beeper registered 3,000 HU. As expected, the capacitor registered lower Hounsfield units than that of the battery, since the capacitor is nearly 90% aluminum (Z=13). It is also expected that the HU magnitude be larger for the battery than that of the capacitor similarly.

Though all the defibrillators studied use the same high voltage capacitor type, not all devices use the same battery. There are four different battery configurations used by devices in this study. The Adapta model ADDR01 IP battery has a composition that is 56.3% iodine (Z=53) and 28.0% iron (Z=26). The IP battery volume is smaller than that of the ICD battery at merely 3.6 cc for the IP and 7 cc for the ICD. For other devices, more titanium (Z=22) may be included in the construction of their batteries. Although smaller, the pacemaker battery registered a greater value at 8,000 HU. Abutting the battery, a 0.8 mm thick nearly pure copper (Z=29) winded telemetry antenna registering 15,000–20,000 HU was also seen in the pacemaker.

In Fig. 4, each area of high Z concentration is illustrated. For the ICD on top, it is now clear that more dose attenuation was observed on the anterior side primarily because of the construction, with the battery located more superior to that of the capacitor. Additionally, it is also observed that the lateral scatter points A→C represent the greatest change possible, as their location is adjacent to that battery. For the pacemaker on the bottom, greater attenuation was visualized in the treatment planning computer on the right side, as illustrated. This is observed by the right‐sided placement of the battery for that device. It is expected similarly that the highest change in lateral scatter be calculated through points J→L where they are more proximal to the battery as well. Although the battery of the ICD was thicker, it exhibited less attenuation comparatively, due to the higher atomic number of the battery from the pacemaker.

It was conclusive from the superior‐inferior calculation points of the ICD that significant attenuation is seen in the treatment planning process. An example of this is shown in Fig. 6. In this view, the attenuation is more noticeable for the pacemaker between isodose levels at 83% (mostly flat) and 78% (tilted superior‐inferiorly). As compared to Fig. 4, points L→J lie beneath the battery, whereas points O→M lie beneath the capacitor. Attenuation from the 6 MV beam for the Maximo DRTM were calculated to be −9.8% at point L and −6.1% at point O. Deeper in the phantom, the attenuation was calculated to be −8.0% at point J and −5.5% at point M. Thus, limited dose differences were identified between planes 0.5 cm and 4.0 cm from the device. In the superior‐inferior direction, the gradient was 3.7% at the plane 0.5 cm from the device, and 2.5% at the plane 4.0 cm deeper from it.

These effects may be observed additionally by considering the dose gradient change in the anterior‐posterior direction. The dose gradient between points J→L is +1.8%, whereas the dose gradient between points O→M is only +0.6%. Thus, at distances further away from the device, a shift in the isodose distribution downward and more anterior is noticeable. This is consistent for all devices with the high Z battery positioned anterior in the device and with more beam attenuation from it.

## V. CONCLUSIONS

When presented with a task to treat patients that have been prescribed an implantable cardioverter‐defibrillator or implantable pacemaker, many options exist for radiation oncologists to consider. Four of these are to: (1) decline to treat altogether at the risk of causing detriment to the device; (2) treat only after communication with the implanting physician with agreed consideration to remove the device temporarily and prior to irradiation; (3) treat with the device intact while integrating ordinary geometries and with a reduced prescription dose to satisfy the device dose limit; or (4) treat with the device intact but utilizing non‐ideal beam arrangements to avoid the device entirely.^(30)^ The third option is least likely the choice, since tumor dose prescriptions currently far exceed device dose limitations. The latter option is typically chosen, although it may result in distributions of dose that are not optimal for the target disease, and higher doses to normal tissue that could have been avoided otherwise. Having a higher dose tolerance for implantable pacemakers and implantable cardioverter‐defibrillators will be an important milestone to achieve. It is this maturity in heart devices that may soon permit us to implement better geometries for treating lung cancers and other chest diseases.

It has been of recent interest to pursue methods of improving upon the functionality and stability of implantable pacemakers and cardioverter‐defibrillators. These efforts have led at least one manufacturer to design a device with MRI compatibility, currently in FDA monitored clinical trials. Due to further advances with regard to efficient battery power consumption and radiation hardened electrical circuits, an increased interest is now being seen with regard to radiation therapy dose effects when a device is near or directly in the field of radiation. This research, which has never been addressed in medical physics to date, presents results from dose delivery simulation using a treatment planning computer. An extensive set of implantable pacemakers and cardioverter‐defibrillators have been studied, with measurements included to validate these simulation studies, constituting results for 32% of all device models marketed by any company nationally.

This research provides a sound basis for direct comparison of simulated radiation therapy dosimetric changes exhibited in patients having been implanted with a pacemaker or defibrillator. Variances and perceived complexities seen from computer modeling in comparison to radiation measurements performed were presented. As meticulously carried out here, identifying the internal structure of devices can aid in the understanding of abnormal dose simulation observances, which could be important clinically.

The first comparison of dosimetry effects from implantable cardoverter‐defibrillators and pacemakers in a therapeutic beam of radiation was described here. Results are concluded on the strict basis of direct radiation measurement. Attenuation, backscatter, and lateral scatter were determined to be −13.4%, 2.1% and 1.5% at 6 MV, and −6.1%, 3.1% and 5.1% at 18 MV, for the ICD group. For the IP group, this research concluded results of −15.9%, 2.8% and 2.5% at 6 MV, and −9.4%, 3.4% and 5.7% at 18 MV. These results were determined accurate within a deviation of only ± 1.8% as deduced from simulation statistics. For IP and ICD leads, measured dose changes were less than 4%, existing as attenuation processes only, and invariant with regard to x‐ray energy. The research concludes with a greater scientific understanding of device interaction with high energy X‐ray beams used for radiation oncology patients.

## ACKNOWLEDGEMENTS

The opportunity to study each of the implantable pacemakers and implantable cardioverter‐defibrillators was supported by the issuance of Medtronic, Inc. Grant # 1106 (to MSG). We would also like to thank Thomas M. Kraus, B.S. of CNMC Company, Inc. for rapid turn‐around time in ADCL calibration of the model 206 electrometer.
